# Perspectives and challenges of multidisciplinary collaboration in the treatment of metastatic spinal disease: Insights from an international survey

**DOI:** 10.1093/nop/npaf102

**Published:** 2025-09-26

**Authors:** Jantijn J G J Amelink, Bram T van Munster, Olivier Q Groot, Britt B M Suelmann, Philip J Saylor, Nicolien Kasperts, Shannon MacDonald, Mark H Bilsky, Jorrit-Jan Verlaan, Daniel G Tobert

**Affiliations:** Department of Radiation Oncology, University Medical Center Utrecht, Utrecht, The Netherlands (J.J.G.J.A., B.T.V.M., N.K., J.-J.V.); Department of Orthopaedic Surgery, Massachusetts General Hospital—Harvard Medical School, Boston, Massachusetts, United States (J.J.G.J.A., B.T.V.M., D.G.T.); Department of Radiation Oncology, University Medical Center Utrecht, Utrecht, The Netherlands (J.J.G.J.A., B.T.V.M., N.K., J.-J.V.); Department of Orthopaedic Surgery, Massachusetts General Hospital—Harvard Medical School, Boston, Massachusetts, United States (J.J.G.J.A., B.T.V.M., D.G.T.); Department of Orthopaedic Surgery, University Medical Center Utrecht, Utrecht, The Netherlands (O.Q.G.); Department of Medical Oncology, University Medical Center Utrecht, Utrecht, The Netherlands (B.B.M.S.); Mass General Cancer Center, Massachusetts General Hospital—Harvard Medical School, Boston, Massachusetts, United States (P.J.S.); Department of Radiation Oncology, University Medical Center Utrecht, Utrecht, The Netherlands (J.J.G.J.A., B.T.V.M., N.K., J.-J.V.); Department of Radiation Oncology, Massachusetts General Hospital—Harvard Medical School, Boston, Massachusetts, United States (S.M); Department of Neurosurgery, Memorial Sloan Kettering Cancer Center, New York, New York, United States (M.H.B.); Department of Radiation Oncology, University Medical Center Utrecht, Utrecht, The Netherlands (J.J.G.J.A., B.T.V.M., N.K., J.-J.V.); Department of Orthopaedic Surgery, Massachusetts General Hospital—Harvard Medical School, Boston, Massachusetts, United States (J.J.G.J.A., B.T.V.M., D.G.T.)

**Keywords:** decision making, interdisciplinary therapeutic approaches, metastatic spinal disease, multidisciplinary collaboration, regional care pathways

## Abstract

**Abstract:**

BackgroundAdvances in systemic therapies, radiotherapy, and surgery have made the management of metastatic spinal disease (MSD) more complex, increasing the need for multidisciplinary collaboration. This study aims to explore physicians’ perspectives on multidisciplinary collaboration and to identify challenges in delivering multidisciplinary care for patients with MSD.

**Methods:**

A cross-sectional, global, internet-based survey was distributed among medical oncologists, radiation oncologists, orthopedic surgeons, and neurosurgeons involved in the management of MSD. Questions focused on the effectiveness of current multidisciplinary collaboration, outcomes of multidisciplinary collaboration, and barriers to providing multidisciplinary care.

**Results:**

A total of 120 physicians completed the survey: 26 (22%) medical oncologists, 34 (28%) radiation oncologists, 31 (26%) orthopedic surgeons, and 29 (24%) neurosurgeons. Across all four specialties, approximately 75% considered current collaboration among medical oncologists, radiation oncologists, and spine surgeons effective. In total, 49% of respondents observed cases in which preventable complications occurred due to a perceived lack of multidisciplinary collaboration, and 69% reported observing cases where a lack of multidisciplinary collaboration resulted in the selection of suboptimal treatment strategies. Overall, respondents reported 162 challenges in delivering multidisciplinary care for patients with MSD, most commonly citing limited regional coordination between hospitals (28%) and insufficient collaboration among specialties (18%).

**Conclusions:**

Although most respondents viewed current multidisciplinary collaboration as effective, many reported observing cases where a lack of collaboration led to preventable complications or suboptimal care. These findings highlight the importance of strengthening multidisciplinary collaboration and enhancing regional coordination to improve outcomes for patients with MSD.

Key Points

- Inadequate multidisciplinary collaboration can result in preventable complications and suboptimal treatment.
- Limited regional coordination and insufficient interspecialty collaboration hinder the delivery of effective multidisciplinary care.
- Strengthening multidisciplinary collaboration, enhancing regional coordination, and adopting shared decision-making are essential for optimizing outcomes.

Importance of the Study

While prior research has emphasized the clinical complexity of managing metastatic spinal disease and the need for integrated multidisciplinary care, few studies have examined the practical challenges and perceived consequences of insufficient collaboration across specialties. This study reveals that preventable complications and suboptimal treatment strategies occur relatively frequently due to poor collaboration between specialties. It also identifies key barriers to effective multidisciplinary care, including limited regional coordination and insufficient collaboration between specialists. Despite limitations related to response bias and regional representation, this study underscores the need to strengthen multidisciplinary collaboration and enhance regional coordination to improve outcomes for patients with metastatic spinal disease.

The prevalence of metastatic spinal disease (MSD) is increasing due to an aging population and improved overall survival rates for patients living with cancer.^1^ Recent advancements in systemic oncological therapy, radiotherapy, and surgery have expanded treatment options for MSD and facilitated the development of combined treatment approaches.^2^ As a result, in certain patients with a life ­expectancy justifying surgical intervention, the management of MSD has become increasingly complex and often requires coordination of systemic therapy, radiotherapy, and sometimes surgery to address symptoms caused by both mechanical and biological tumor factors.^3^

Multidisciplinary collaboration refers to specialists from different disciplines working collectively and contributing their expertise to achieve a common goal amid uncertainty and complexity.^4^ In the context of MSD, multidisciplinary collaboration among medical oncologists, radiation oncologists, and spine surgeons has been shown to play a crucial role in timely referral, treatment decision-making, and managing patient expectations and satisfaction.^5^^,^^6^ Furthermore, combining different treatment modalities, such as systemic therapy, radiotherapy, and surgery requires careful consideration. Complications may arise when treatments are not properly coordinated (eg, in terms of sequencing or timing), including impaired postoperative wound healing, postoperative infections, delays in initiating or resuming systemic therapy, and failure to achieve local tumor control.^3^ Despite the growing recognition of the need for multidisciplinary collaboration in managing MSD, the perspectives of various medical specialists involved remain largely unexplored.^7^

To address this gap, we conducted a survey of medical oncologists, radiation oncologists, orthopedic surgeons, and neurosurgeons. This survey aimed to explore perspectives on multidisciplinary collaboration and to identify challenges in delivering multidisciplinary care for patients with MSD. The study findings represent an initial step toward identifying priority areas for coordinating resources and policies to enhance multidisciplinary care for patients with MSD.

## Methods

### Study Setting and Survey Development

Our institutional review board approved this international cross-sectional survey to examine physician perspectives on multidisciplinary collaboration in the treatment of MSD (registration: 2023P002834). The survey was developed by physicians representing various medical specialties that play a considerable role in the decision-making process, specifically medical oncologists (B.B.M.S. and P.J.S.), radiation oncologists (N.K. and S.M.), and spine surgeons (J.J.V. and D.G.T.). These collaborating physicians formulated ­questions, reviewed them for validity and relevance, and refined them for clarity. The survey was conducted in compliance with the principles of the Declaration of Helsinki (2013) and the General Data Protection Regulation (2018).^8^^,^^9^ The survey was designed to be completed in less than ten minutes and responses were collected anonymously. Data collection and survey distribution were performed using the *REDCap* platform. The Checklist for Reporting of Survey Studies (CROSS) was followed (Appendix A).^10^

### Survey Distribution

A link to the survey was distributed to all members of the Dutch Spine Society (DSS), the North American Spine Society (NASS) section on spinal oncology, the Dutch National Platform for Palliative Radiotherapy (NPPR), and to medical oncologists within the affiliated institutions of the authors.^11^ Since the authors were based in the Netherlands and the United States, the survey was primarily distributed to treating physicians through networks in these two regions. Due to the open-ended nature of the survey distribution, the response rate among members of these societies could not be assessed. Additionally, the survey was shared with various specialists within the authors’ professional network, including medical oncologists, radiation oncologists, orthopedic surgeons, and neurosurgeons. Respondents were encouraged to further disseminate the survey by inviting colleagues and acquaintances involved in the management of MSD. Participation in the survey was anonymous, voluntary, and without remuneration. Complete survey responses were accepted from November 1, 2023 to April 30, 2024.

### Measures

The survey consisted of 29 questions designed to evaluate the following four domains: (1) demographics and professional characteristics of respondents (*n* = 6); (2) attitudes toward multidisciplinary collaboration (*n* = 13); (3) impact of multidisciplinary collaboration (*n* = 8); and (4) challenges in providing multidisciplinary care (*n* = 2) (Appendix B). Respondents were asked to what extent they felt there is effective multidisciplinary collaboration among treating physicians, specifically medical oncologists, radiation oncologists, and spine surgeons, and whether they believed additional multidisciplinary collaboration is needed. Other questions included: the number of observed cases where preventable complications occurred due to a lack of multidisciplinary collaboration, the number of observed cases where suboptimal treatment strategies were selected due to a lack of multidisciplinary collaboration, and challenges encountered in providing multidisciplinary care for patients with MSD. For some questions (eg, type of primary healthcare practice, defined as the hospital or facility where respondents provide the majority of patient care) categories were mutually exclusive, but for other questions (eg, challenges in providing multidisciplinary care) multiple responses were allowed. In the latter case, each response option was treated as a separate dichotomous variable.

### Statistical Analysis

Categorical and dichotomous variables were summarized using frequencies and percentages. Differences in demographic and professional characteristics were evaluated using Fisher’s exact test. Descriptive statistics were reported overall, reflecting all respondents, and stratified by specialty: medical oncologists, radiation oncologists, orthopedic surgeons, and neurosurgeons. For ordinal categorical variables, differences between specialties were assessed using the Kruskal-Wallis test.^12^  *Post hoc* pairwise comparisons were performed using pairwise Fisher’s exact tests with Bonferroni correction for non-ordinal categorical variables and Dunn’s test with Bonferroni correction for ordinal categorical variables.^13^  *P*-values <.05 were considered statistically significant. Statistical analyses were performed using RStudio version 4.3.1 (2023-06-16 UCRT).

## Results

### Respondent Characteristics

A total of 120 complete survey responses were collected, including 26 medical oncologists (22%), 34 radiation oncologists (28%), 31 orthopedic surgeons (26%), and 29 neurosurgeons (24%). The majority of survey respondents (*n* = 118; 98%) had undergone specialty training and practice in either the European region or the North American region (United States/Canada). Radiation oncologists more often underwent specialty training and practice in Europe, while medical oncologists more often underwent specialty training and practice in the North American region (*P* < .05). Fifty-eight percent of respondents (*n* = 70) had more than 10 years of experience as an attending physician treating patients with MSD, while 42% of respondents (*n* = 50) had 10 years or less of experience. Survey respondents were primarily employed in academic/university hospitals (*n* = 93; 78%). Medical oncologists almost exclusively practice in academic/university hospitals, whereas radiation oncologists relatively more often practice in public or government/military hospitals (*P* < .05; Table 1).

**Table 1.** Demographic and professional characteristics of survey respondents by specialty

Respondent characteristics	Radiation oncologists	Orthopedic surgeons	Neurosurgeons	Medical oncologists	*P*-value
Number of respondents	34	31	29	26	
Specialization training region					**<.05**
European Region	**27 (79%)^↑^**	12 (39%)	17 (59%)	**5 (19%)^↓^**	
American Region—United States/Canada	**6 (18%)^↓^**	19 (61%)	11 (38%)	**21 (81%)^↑^**	
American Region—Latin America	1 (3%)	0 (0%)	0 (0%)	0 (0%)	
South-east Asian Region	0 (0%)	0 (0%)	1 (3%)	0 (0%)	
Experience as attending physician					.72
<5 years	5 (14%)	4 (13%)	7 (24%)	3 (12%)	
5-10 years	8 (24%)	11 (36%)	6 (21%)	6 (23%)	
11-15 years	8 (24%)	6 (19%)	7 (24%)	4 (15%)	
16-20 years	8 (24%)	4 (13%)	2 (7%)	6 (23%)	
>20 years	5 (14%)	6 (19%)	7 (24%)	7 (27%)	
Number of patients with symptomatic spinal metastases treated annually					.07
1-9 patients	1 (3%)	7 (22%)	7 (24%)	10 (38%)	
10-20 patients	11 (32%)	8 (26%)	5 (17%)	10 (38%)	
21-40 patients	12 (35%)	8 (26%)	10 (35%)	3 (12%)	
41-60 patients	5 (14%)	3 (10%)	2 (7%)	1 (4%)	
61-80 patients	2 (6%)	3 (10%)	0 (0%)	1 (4%)	
>80 patients	3 (10%)	2 (6%)	5 (17%)	1 (4%)	
Type of primary healthcare practice					**<.05**
Academic/University hospital	23 (68%)	24 (77%)	21 (72%)	**25 (96%)^↑^**	
Public hospital or Government/Military hospital	**10 (29%)^↑^**	4 (13%)	6 (21%)	0 (0%)	
Private practice	0 (0%)	3 (10%)	2 (7%)	0 (0%)	
Other	1 (3%)	0 (0%)	0	1 (4%)	
Region of practice					**<.05**
European Region	**27 (79%)^↑^**	12 (39%)	17 (59%)	**4 (15%)^↓^**	
American Region—United States/Canada	**7 (21%)^↓^**	18 (58%)	11 (38%)	**22 (85%)^↑^**	
American Region—Latin America	0 (0%)	0 (0%)	0 (0%)	0 (0%)	
South-east Asian Region	0 (0%)	1 (3%)	1 (3%)	0 (0%)	

Fisher exact test was performed for all categorical variables.

**Bold**  *P*-values indicate statistical significance of *P* < .05.

**Bold** counts and percentages, along with arrows (↑ and ↓), indicate categories with significantly higher (↑) or lower (↓) proportions compared to other specialties, based on post-hoc pairwise Fisher’s exact tests with Bonferroni correction (*P* < .05).

### Attitudes Toward Multidisciplinary Collaboration

**Figure 1.**
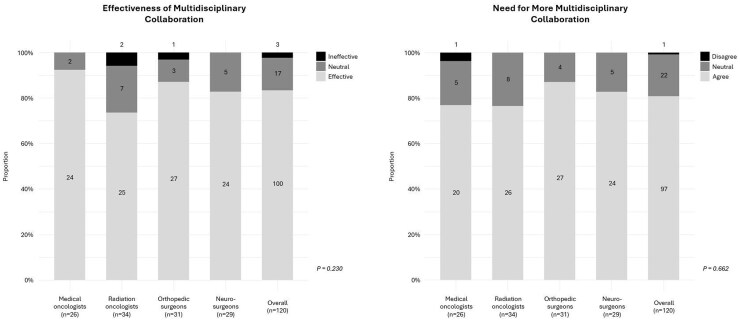
Responses by specialty and overall on current effectiveness and the need for increased multidisciplinary collaboration.

The majority of medical oncologists (*n* = 24; 92%), radiation oncologists (*n* = 25; 74%), orthopedic surgeons (*n* = 27; 87%), and neurosurgeons (*n* = 24; 83%) reported feeling that multidisciplinary collaboration among treating physicians was “effective” at their respective primary healthcare institutions. Overall, 100 respondents (83%) reported that collaboration was “effective”, 17 respondents (14%) reported feeling “neutral”, and 3 respondents (3%), including 2 radiation oncologists and 1 orthopedic surgeon, reported that multidisciplinary collaboration was “ineffective”. No differences were observed between specialties in their reported effectiveness of multidisciplinary collaboration (*P* = .230). The majority of medical oncologists (*n* = 20; 77%), radiation oncologists (*n* = 26; 77%), orthopedic surgeons (*n* = 27; 87%), and neurosurgeons (*n* = 24; 83%) “agreed” that more multidisciplinary collaboration is needed for the treatment of patients with MSD. Overall, 97 respondents (81%) “agreed” with the need for more multidisciplinary collaboration, 22 respondents (18%) reported feeling “neutral”, and 1 respondent (0.8%), a medical oncologist, disagreed that more multidisciplinary collaboration is needed. No differences were observed between specialties in their reported need for more multidisciplinary collaboration (*P* = .662; Figure 1).

### Impact of Multidisciplinary Collaboration

Nearly half of the respondents (*n* = 59; 49%) reported observing cases where a lack of multidisciplinary collaboration resulted in preventable complications for patients with MSD (eg, impaired postoperative wound healing, delays in initiating or resuming systemic therapy, and failure to achieve local tumor control). Overall, 45 respondents (37.5%) reported observing preventable complications in a few cases (1%-25%), 13 respondents (10.8%) reported such complications in some cases (26%-50%), and 1 respondent (0.8%) reported that preventable complications occurred in many cases (51%-75%). A total of 61 respondents (51%) reported that they had not observed any relevant cases in which preventable complications occurred due to a perceived lack of multidisciplinary collaboration. Differences in responses were observed between specialties (*P* = .043), with pairwise comparisons revealing that orthopedic surgeons (*P* = .014) reported observing preventable complications in patients with MSD more frequently than medical oncologists. No other differences were found when comparing the remaining groups (all *P* > .05).

**Figure 2.**
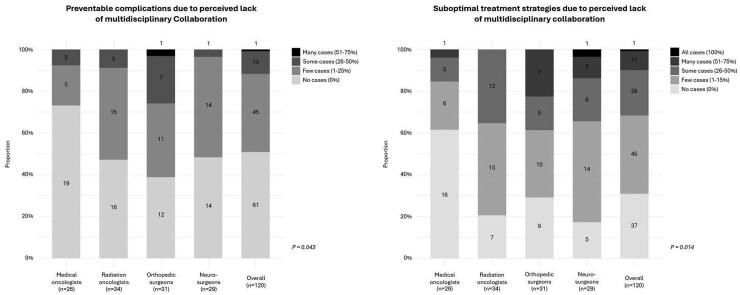
Responses by specialty and overall on the incidence of preventable complications and suboptimal treatment strategies due to a lack of multidisciplinary collaboration.

More than two-thirds of respondents (*n* = 83; 69.2%) reported observing cases where a lack of multidisciplinary collaboration resulted in the selection of suboptimal treatment strategies for patients with MSD. Overall, 45 respondents (37.5%) reported observing suboptimal treatment strategies in few cases (1%-25%), 26 respondents (21.7%) reported such strategies in some cases (26%-50%), 11 respondents (9.2%) reported that suboptimal treatment strategies were selected in many cases (51%-75%), and 1 respondent (0.8%) reported that suboptimal treatment strategies were selected in all cases (100%). A total of 37 respondents (30.8%) reported that they had not observed any relevant cases in which a lack of multidisciplinary collaboration resulted in the selection of suboptimal treatment strategies. Differences in responses were observed between specialties (*P* = .014), with pairwise comparisons revealing that radiation oncologists (*P* = .022), orthopedic surgeons (*P* = .013), and neurosurgeons (*P* = .007) each reported observing suboptimal treatment strategies for patients with MSD more frequently than medical oncologists. No other differences were found when comparing the remaining groups (all *P* > .05; Figure 2). Subgroup analyses by region (European region vs. American region) and by experience as an attending physician (≤10 years vs. >10 years) also found no differences in reported preventable complications or suboptimal treatment strategies (Appendix C).

### Challenges in Providing Multidisciplinary Care

**Figure 3.**
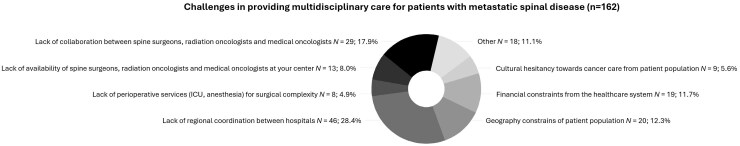
Overall responses on challenges in providing multidisciplinary care for patients with metastatic spinal disease.

In total, 109 respondents (91%) reported 162 challenges they encountered in providing multidisciplinary care for patients with MSD. The most frequently mentioned challenges were lack of regional coordination between hospitals (*n* = 46; 28.4%) and lack of collaboration among medical oncologists, radiation oncologists, and spine surgeons (*n* = 29; 17.9%). Furthermore, respondents reported other challenges 18 times (11.1%), with time constraints for multidisciplinary team meetings being the most frequently mentioned. A total of 11 respondents (9%) reported that they did not encounter any challenges in providing multidisciplinary care (Figure 3).

## Discussion

To our knowledge, this is the first survey to explore perspectives on multidisciplinary collaboration and the challenges of providing multidisciplinary care for patients with MSD. We found that attitudes toward multidisciplinary collaboration were largely consistent across medical oncologists, radiation oncologists, orthopedic surgeons, and neurosurgeons. Notably, respondents reported preventable complications and suboptimal treatment strategies resulting from a lack of multidisciplinary collaboration in a substantial number of cases. Additionally, nearly all respondents reported encountering several challenges in providing multidisciplinary care for patients with MSD. These survey data provide a critical initial assessment of physicians’ perspectives on multidisciplinary collaboration and highlight key areas to further enhance care for patients with MSD.

### Importance of Multidisciplinary Collaboration

Prolonged survival in patients with MSD has increased the need for durable palliation and long-term disease control.^14^ Concurrent advancements in surgical techniques, radiotherapy, and systemic therapies have expanded treatment options, transforming treatment decisions from relatively straightforward choices between surgery and radiation into complex, multimodal evaluations that require integrated strategies.^15^^,^^16^ Taken together, these developments underscore the critical role of multidisciplinary collaboration in the contemporary management of MSD. Our survey found that physicians across all four specialties currently perceive multidisciplinary collaboration as effective in their primary healthcare settings, while also expressing a strong need for increased collaboration. This apparent discrepancy may be partly explained by the tendency of respondents to provide socially desirable responses, acknowledging the effectiveness of current multidisciplinary collaboration while simultaneously recognizing the need for improvement.^17^ However, the expressed need for increased collaboration is in line with prior research, particularly the work by Barton et al.^18^ where treating clinicians identified multidisciplinary collaboration as one of the most important factors in treatment decision-making for patients with spinal metastases. Given the increased complexity of managing MSD, it is understandable that physicians recognize the (relative) effectiveness of current multidisciplinary efforts while emphasizing the need for greater collaboration to keep pace with the evolving treatment landscape.^19^

### Insufficient Multidisciplinary Collaboration

Combining different treatment modalities from different disciplines can improve symptom management and reduce morbidity in patients with MSD.^20^ Nevertheless, complications may arise if treatments are not carefully coordinated in terms of sequencing, timing, and integration.^3^ For example, performing surgery and postoperative radiotherapy in quick succession can impair wound healing and increase the risk of surgical site infections, potentially delaying the initiation or resumption of systemic therapy.^21^ Similarly, administering certain systemic therapies around the time of surgery can adversely affect tissue healing and delay subsequent postoperative radiotherapy, thereby increasing the risk of early local recurrence.^22–24^ In the present study, nearly half of respondents reported observing complications due to inadequate multidisciplinary collaboration. Interestingly, orthopedic surgeons more often perceived such complications compared with medical oncologists, which may partly reflect their more direct role in managing surgical complications such as wound healing problems and surgical site infections. These findings are particularly relevant, as complications are relatively common in patients with MSD and can significantly impact quality of life.^25–27^ Preventing them is therefore essential. In addition, more than two-thirds of respondents reported observing cases where a lack of multidisciplinary collaboration resulted in the selection of suboptimal treatment strategies. Radiation oncologists, orthopedic surgeons, and neurosurgeons reported such observations more frequently than medical oncologists. This likely reflects their greater visibility of suboptimal care, such as delays in surgery or radiotherapy, whereas medical oncologists may be less exposed to these procedural consequences. These findings are in line with a recent study reporting that approximately 30% of patients with spinal metastases received an alternative strategy despite meeting the criteria for separation surgery according to the NOMS framework.^28^ Collectively, these findings highlight the clinical importance of multidisciplinary collaboration in guiding the selection of optimal treatment strategies that are generally less invasive, have lower surgical morbidity, and better preserve quality of life.^29^^,^^30^

### Challenges in Providing Multidisciplinary Care

Although respondents generally perceive multidisciplinary collaboration as effective, they encounter several challenges in providing multidisciplinary care for patients with MSD. The two challenges reported most frequently were limited regional coordination between hospitals and insufficient collaboration among medical oncologists, radiation oncologists, and spine surgeons. In other oncology settings, research has shown that implementing regional oncology networks can enhance collaboration and coordination across hospitals, thereby improving patient outcomes and satisfaction.^31^^,^^32^ Establishing similar initiatives for patients with MSD could optimize multidisciplinary care and enhance outcomes and patient experience. Future efforts should therefore prioritize developing regional oncology networks to strengthen collaboration and lay the foundation for regionally organized multidisciplinary care for patients with MSD. Furthermore, to address insufficient collaboration between specialties, efforts should focus on facilitating efficient and accessible communication among treating physicians and promoting multidisciplinary approaches to treatment decision-making. Potential strategies include regular multidisciplinary team meetings and multidisciplinary clinics, which could improve coordination, reduce treatment delays, and ensure fully informed, collaborative treatment decision-making.

### Implications for Clinical Practice

This survey demonstrates that, while physicians across all four specialties generally consider multidisciplinary collaboration to be effective, a lack of collaboration contributes to complications and suboptimal treatment strategies. Additionally, respondents reported encountering several challenges in providing multidisciplinary care for patients with MSD. These findings highlight the need to further strengthen collaboration between specialties and the delivery of multidisciplinary care for patients with MSD. Next steps should focus on measures that enhance collaboration and coordination, such as establishing multidisciplinary clinics and developing regional oncology networks. Furthermore, patients and their advocates should be informed that, according to physicians involved in the management of MSD, multidisciplinary collaboration is associated with improved outcomes. Providing tools to support patient engagement may empower patients and their advocates to ask informed questions about multidisciplinary communication, monitor coordination of care, and participate actively in shared decision-making.

### Limitations

This study has several limitations. First, due to the nature of the survey distribution, we were unable to determine the exact response rate, which may have introduced response bias, leading to an overrepresentation of positive results. Second, as the survey was conducted anonymously, no measures were in place to identify or prevent potential duplicate responses, which could affect data integrity. Third, as the authors are based in the Netherlands and the United States, 98% (118/120) of respondents completed their specialization training and practice in either Europe or the North American region (United States/Canada), potentially limiting the generalizability of our findings. Also, given the global variability in the state and challenges of multidisciplinary collaboration, these findings may not be applicable to all healthcare settings. Fourth, the survey did not include input from other essential subspecialties, such as general practitioners, pain specialists, interventional radiologists, and rehabilitation physicians, whose perspectives are crucial in improving multidisciplinary care for patients with MSD. General practitioners, in particular, warrant inclusion, given their key role in early symptom recognition and timely referral.^6^ Finally, there is a potential risk of confirmation bias, as physicians who already value the importance of multidisciplinary collaboration may have been more likely to participate, which could lead to an overestimation of the perceived challenges and needs in this area. However, given the broad representation across various specialties, it is likely that the impact of this bias is limited, and our findings still offer valuable insights into the perspectives of physicians involved in the management of MSD. Nevertheless, these limitations highlight the need for future research with larger sample sizes, prospective data collection, and inclusion of respondents from a broader range of regions. In particular, studies should incorporate the perspectives of additional subspecialties, including general practitioners and rehabilitation physicians, to better reflect real-world practice and identify further opportunities to improve multidisciplinary care for patients with MSD.

This study is an exploratory investigation aimed at describing the perspectives and challenges faced by medical oncologists, radiation oncologists, orthopedic surgeons, and neurosurgeons in providing multidisciplinary care for patients with MSD. It was not designed nor powered to detect independent relationships or statistical significance. Therefore, the inability to detect statistically significant differences, even in the presence of clinically meaningful variations, should be interpreted with caution. Despite our best efforts, the findings may primarily reflect the perspectives of physicians in Europe and the North America. However, given the relatively large number of respondents and the absence of financial incentives for participation, we are confident that our survey serves as a starting point for further shaping and coordinating multidisciplinary care for patients with MSD, ultimately enhancing the quality of care for this medically fragile patient population.

## Conclusion

This survey underscores the importance of multidisciplinary collaboration in managing patients with MSD. While physicians across multiple specialties generally perceive current multidisciplinary collaboration as effective, a lack of collaboration contributes to preventable complications and suboptimal treatment strategies. Reported challenges in delivering multidisciplinary care included limited regional coordination between hospitals and insufficient collaboration among specialists. Continuous efforts are needed to improve communication and collaboration among treating physicians, strengthen regional coordination of care, and promote a shared multidisciplinary approach to treatment decision-making. This survey provides a foundation for further development and coordination of multidisciplinary care for patients with MSD. Future studies should include additional subspecialties and prospective data to better understand the delivery, scope, and impact of multidisciplinary care on patient outcomes.

## Supplementary Material

npaf102_Supplementary_Data

## Data Availability

The datasets generated during and/or analyzed during the current study are not publicly available due to legal restrictions arising from data-sharing agreements but are available from the corresponding author on reasonable request.
