# Supplementary material for: Perspectives and challenges of multidisciplinary collaboration in the treatment of metastatic spinal disease: Insights from an international survey
Source: Neurooncol Pract. 2025 Sep 26;13(2):322–30. doi: 10.1093/nop/npaf102 (PMC13153715; doi:10.1093/nop/npaf102)
Supplement: npaf102_Supplementary_Data [file npaf102_supplementary_data.zip › npaf102_Supplementary_Data/Appendix_B.docx]

**Appendix B. Survey**

**1. Demographics and professional characteristics of respondents (n=6):**

What is your specialization?

- Neurosurgery
- Orthopedic surgery
- Medical oncologists
- Radiation oncologists
- other

In which region did you complete your specialization training?

- American Region – United States/Canada
- American Region – Latin America
- European Region
- Eastern Mediterranean Region
- South-east Asian Region
- Western Pacific Region
- African region

How many years have you been working as a spine surgeon/medical oncologist/radiation oncologist?

- < 5 years
- 5-10 years
- 11-15 years
- 16-20 years
- 20 years

How many patients with symptomatic spinal metastases do you treat each year?

- 0 patients
- 1-9 patients
- 10-20 patients
- 21-40 patients
- 41-60 patients
- 61-80 patients
- 80 patients

In which type of healthcare practice do you primarily provide treatment for patients with symptomatic spinal metastases? *We define an academic hospital as one that is directly affiliated with a research university

- Academic/University hospital*
- Public hospital or Government/Military hospital
- Private practice
- Other

What is the region of your primary practice?

- American Region – United States/Canada
- American Region – Latin America
- European Region
- Eastern Mediterranean Region
- South-east Asian Region
- Western Pacific Region
- African region

**2. Attitudes towards multidisciplinary collaboration (n=13):**

Following questions were asked if Q1 was neurosurgery or orthopedic surgery.

Do you ever consult with a radiation oncologist before operating on a patient with symptomatic spinal metastases?

- Yes
- No

In what percentage of these patients do you consult with a radiation oncologist before operating?

- All patients (100%)
- Most patients (76-99%)
- Many patients (51-75%)
- Some patients (26-50%)
- Few patients (1-25%)

Do you ever consult with a medical oncologist before operating on a patient with symptomatic spinal metastases?

- Yes
- No

In what percentage of these patients do you consult with a medical oncologist before operating?

- All patients (100%)
- Most patients (76-99%)
- Many patients (51-75%)
- Some patients (26-50%)
- Few patients (1-25%)

Following questions were asked if Q1 was medical oncologist.

Do you ever consult with a radiation oncologist before initiating systemic oncologic therapy for a patient with symptomatic spinal metastases?

- Yes
- No

In what percentage of these patients do you consult with a radiation oncologist before initiating systemic oncologic therapy?

- All patients (100%)
- Most patients (76-99%)
- Many patients (51-75%)
- Some patients (26-50%)
- Few patients (1-25%)

Do you ever consult with a spine surgeon before initiating systemic oncologic therapy for a patient with symptomatic spinal metastases?

- Yes
- No

In what percentage of these patients do you consult with a spine surgeon before initiating systemic oncologic therapy?

- All patients (100%)
- Most patients (76-99%)
- Many patients (51-75%)
- Some patients (26-50%)
- Few patients (1-25%)

Following questions were asked if Q1 was radiation oncologist.

Do you ever consult with a spine surgeon before initiating radiotherapy on a patient with symptomatic spinal metastases?

- Yes
- No

In what percentage of these patients do you consult with a spine surgeon before initiating radiotherapy?

- All patients (100%)
- Most patients (76-99%)
- Many patients (51-75%)
- Some patients (26-50%)
- Few patients (1-25%)

Do you ever consult with a medical oncologist before initiating radiotherapy on a patient with symptomatic spinal metastases?

- Yes
- No

In what percentage of these patients do you consult with a medical oncologist before initiating radiotherapy?

- All patients (100%)
- Most patients (76-99%)
- Many patients (51-75%)
- Some patients (26-50%)
- Few patients (1-25%)

Which of the various treatment regimens for patients with symptomatic spinal metastases can be performed without the need to transfer them to different hospitals? (please select all that apply):

- Open surgery, including decompression and stabilization
- Minimally invasive surgery, including percutaneous decompression and stabilization
- Conventional radiotherapy (cEBRT, including IMRT and VMAT)
- Stereotactic radiotherapy (SBRT)
- Systemic cancer therapy (including immunotherapy)
- Embolization (pre-operative)
- Other

Are there multidisciplinary team meetings where patients with symptomatic spinal metastases are discussed?

- Yes
- No

What specialties are routinely involved in these multidisciplinary team meetings? (Please select all that apply):

- Neurosurgery
- Orthopedic surgery
- Medical oncology
- Radiation oncology
- Intervention radiology
- Neurology
- Rehabilitation medicine
- Physical therapist
- General practitioner
- Other

How frequently do these multidisciplinary team meetings take place?

- Once a week
- Once every two weeks
- Once a month
- Once every two months
- Other

What percentage of patients with symptomatic spinal metastases are discussed in these multidisciplinary team meetings?

- All patients (100%)
- Most patients (76-99%)
- Many patients (51-75%)
- Some patients (26-50%)
- Few patients (1-25%)

Is there a decision framework used in determining the treatment strategy for patients with symptomatic spinal metastases?

- Yes
- No

What decision framework is used in determining the treatment strategy for patients with symptomatic spinal metastases?

- NOMS framework
- LMNOP framework
- SENO framework
- Other

To what extent do you feel there is effective multidisciplinary collaboration among spine surgeons, radiation oncologists, and medical oncologists for patients with symptomatic spinal metastases?

- Very effective
- Effective
- Neutral
- Ineffective
- Very ineffective

Do you feel more multidisciplinary collaboration among spine surgeons, radiation oncologists, and medical oncologists is needed for the treatment of patients with symptomatic spinal metastases?

- Strongly agree
- Agree
- Neutral
- Disagree
- Strongly disagree

**3. Impact of multidisciplinary collaboration (n=8):**

Have you observed cases where a lack of multidisciplinary collaboration resulted in the selection of suboptimal treatment strategies for patients with symptomatic spinal metastases?

- Yes
- No

In what percentage of these cases did you observe the selection of suboptimal treatment strategies due to a lack of multidisciplinary collaboration?

- All cases (100%)
- Most cases (76-99%)
- Many cases (51-75%)
- Some cases (26-50%)
- Few cases (1-25%)

Have you observed cases where a lack of multidisciplinary collaboration resulted in preventable complications for patients with symptomatic spinal metastases?

- Yes
- No

In what percentage of cases did you observe preventable complications due to a lack of multidisciplinary collaboration?

- All cases (100%)
- Most cases (76-99%)
- Many cases (51-75%)
- Some cases (26-50%)
- Few cases (1-25%)

How valuable do you consider the use of a decision framework in determining the treatment strategy for patients with symptomatic spinal metastases?

- Extremely valuable
- Very valuable
- Moderately valuable
- Slightly valuable
- Not valuable at all

How valuable do you consider multidisciplinary team meetings where patients with symptomatic spinal metastases are discussed?

- Extremely valuable
- Very valuable
- Moderately valuable
- Slightly valuable
- Not valuable at all

Following questions were asked if Q1 was neurosurgery or orthopedic surgery.

How valuable do you consider consultations with radiation oncologists before operating on patients with symptomatic spinal metastases?

- Extremely valuable
- Very valuable
- Moderately valuable
- Slightly valuable
- Not valuable at all

How valuable do you consider consultations with medical oncologists before operating on patients with symptomatic spinal metastases?

- Extremely valuable
- Very valuable
- Moderately valuable
- Slightly valuable
- Not valuable at all

Following questions were asked if Q1 was medical oncologist.

How valuable do you consider consultations with radiation oncologists before initiating systemic oncologic therapy for patients with symptomatic spinal metastases?

- Extremely valuable
- Very valuable
- Moderately valuable
- Slightly valuable
- Not valuable at all

How valuable do you consider consultations with spine surgeons before initiating systemic oncologic therapy for patients with symptomatic spinal metastases?

- Extremely valuable
- Very valuable
- Moderately valuable
- Slightly valuable
- Not valuable at all

Following questions were asked if Q1 was radiation oncologist.

How valuable do you consider consultations with spine surgeons before initiating radiotherapy on patients with symptomatic spinal metastases?

- Extremely valuable
- Very valuable
- Moderately valuable
- Slightly valuable
- Not valuable at all

How valuable do you consider consultations with medical oncologists before initiating radiotherapy on patients with symptomatic spinal metastases?

- Extremely valuable
- Very valuable
- Moderately valuable
- Slightly valuable
- Not valuable at all

**4. Challenges in providing multidisciplinary care (n=2):**

At your primary healthcare practice, what are the challenges in providing multidisciplinary care for patients with symptomatic spinal metastases? (please select all that apply):

- Lack of collaboration between radiation oncologists, medical oncologists and spine surgeons
- Lack of availability of radiation oncologists, medical oncologists and spine surgeons at your center
- Lack of perioperative services (ICU, anaesthesia) for surgical complexity
- Lack of regional coordination between hospitals
- Geography constrains of patient population
- Financial constraints of healthcare system
- Cultural hesitancy towards cancer care from patient population
- Other

What additional topics do you find important for multidisciplinary collaboration in treating patients with symptomatic spinal metastases?

- _________
