# Supplementary material for: Perspectives and challenges of multidisciplinary collaboration in the treatment of metastatic spinal disease: Insights from an international survey
Source: Neurooncol Pract. 2025 Sep 26;13(2):322–30. doi: 10.1093/nop/npaf102 (PMC13153715; doi:10.1093/nop/npaf102)
Supplement: npaf102_Supplementary_Data [file npaf102_supplementary_data.zip › npaf102_Supplementary_Data/Appendix_C.docx]

**Appendix C.**

| **Table 1.**  **Reported incidence of preventable complications and suboptimal treatment strategies by region (European region vs. American Region – United States/Canada) (n=118)** | | | |
| --- | --- | --- | --- |
| **Respondent characteristics** | **European Region** | **American Region – United States/Canada** | ***P-value*** |
| Number of respondents | 60 (51%) | 58 (49%) |  |
| Preventable complications due to perceived lack of multidisciplinary collaboration |  |  | 0.517 |
| No relevant cases observed | 29 (48%) | 32 (55%) |  |
| Few cases (1-25%) | 25 (42%) | 18 (31%) |  |
| Some cases (26-50%) | 6 (10%) | 7 (12%) |  |
| Many cases (51-75%) | 0 (0%) | 1 (2%) |  |
| Most cases (76-99%) | 0 (0%) | 0 (0%) |  |
| All cases (100%) | 0 (0%) | 0 (0%) |  |
| Suboptimal treatment strategies due to perceived lack of multidisciplinary collaboration |  |  | 0.387 |
| No relevant cases observed | 16 (27%) | 21 (36%) |  |
| Few cases (1-25%) | 27 (45%) | 17 (29%) |  |
| Some cases (26-50%) | 12 (20%) | 13 (23%) |  |
| Many cases (51-75%) | 5 (8%) | 6 (10%) |  |
| Most cases (76-99%) | 0 (0%) | 0 (0%) |  |
| All cases (100%) | 0 (0%) | 1 (2%) |  |
| Wilcoxon rank-sum test was performed for all ordinal categorical variables.  **Bold** p-values indicate statistical significance of p<0.05. | | | |

| **Table 2.**  **Reported incidence of preventable complications and suboptimal treatment strategies by experience as an attending physician (≤10 years vs. >10 years) (n=120)** | | | |
| --- | --- | --- | --- |
| **Respondent characteristics** | **≤10 years of experience** | **>10 years of experience** | ***P-value*** |
| Number of respondents | 50 (42%) | 70 (58%) |  |
| Preventable complications due to perceived lack of multidisciplinary collaboration |  |  | 0.792 |
| No relevant cases observed | 26 (52%) | 35 (50%) |  |
| Few cases (1-25%) | 18 (36%) | 27 (39%) |  |
| Some cases (26-50%) | 5(10%) | 8 (11%) |  |
| Many cases (51-75%) | 1 (2%) | 0 (0%) |  |
| Most cases (76-99%) | 0 (0%) | 0 (0%) |  |
| All cases (100%) | 0 (0%) | 0 (0%) |  |
| Suboptimal treatment strategies due to perceived lack of multidisciplinary collaboration |  |  | 0.167 |
| No relevant cases observed | 13 (26%) | 24 (34%) |  |
| Few cases (1-25%) | 20 (40%) | 25 (36%) |  |
| Some cases (26-50%) | 14 (28%) | 12 (17%) |  |
| Many cases (51-75%) | 2 (4%) | 9 (13%) |  |
| Most cases (76-99%) | 0 (0%) | 0 (0%) |  |
| All cases (100%) | 1 (2%) | 0 (0%) |  |
| Wilcoxon rank-sum test was performed for all ordinal categorical variables.  **Bold** p-values indicate statistical significance of p<0.05. | | | |
